# Dysmorphic physical appearance and psychosocial burdens in Klippel-Feil condition

**DOI:** 10.1192/j.eurpsy.2024.1222

**Published:** 2024-08-27

**Authors:** N. Bouayed Abdelmoula, R. Rannen, B. Abdelmoula

**Affiliations:** Genomics of Signalopathies at the service of Precision Medicine - LR23ES07, Medical University of Sfax, Sfax, Tunisia

## Abstract

**Introduction:**

Klippel-Feil abnormality (KFA) is an association of bone defects characterized by a triad: fusion of the cervical vertebrae and consequent short neck, low hairline and a limited motion in the neck. KFA may be a feature of another disorder, such as MURCS association. Familial mutations in the GDF6 (KFS1 8q22), MEOX1 (KFS2 17q21), GDF3 (KFS3 12p13) and MYO18B (KFS4 22q11) genes cause inherited KFA.

**Objectives:**

The aim of this study was to report dysmorphic features and psychological burdens in two sisters with Klippel-Feil condition.

**Methods:**

Two sisters with amenorrhea and dysmorphic clinical features were examined at our genetic counselling. Assessment of dysmorphic and behavioral features and karyotyping using RHG banding were performed.

**Results:**

Familial history revealed consanguineous parents and seven other healthy sisters. Physical examination shown typical triad of KFA. Karyotyping showed 46,XX formula in both patients. The first 22-year-old sister had body asymmetry with size difference between the two sides at the level of bones, pectus excavatum of the sternum, an ascent of the left scapula, scoliosis, dental position abnormalities and facial dysmorphism. The second 28-year-old sister had size difference between the two legs and scoliosis, vitiligo and facial dysmorphism. Anxious and depressed, the two sisters had normal learning abilities but shared many personal psychological concerns regarding their physical appearance and their amenorrhea. They were also exposed to significant discrimination and stigma making them feel excluded and ignored because of their visible difference.

**Conclusions:**

Physical appearance has a profound impact on a person’s life. To our knowledge, there is no reports that describe specific psychological burdens of KFA. Self-esteem, body image, and quality of life is negatively impacted in the case of dysmorphic physical appearance, always associated to social discrimination. Patients with KFA should be assessed not only for associated congenital defects but also for psychological distresses.

**Disclosure of Interest:**

None Declared

